# The effects of six months *Persicaria minor* extract supplement among older adults with mild cognitive impairment: a double-blinded, randomized, and placebo-controlled trial

**DOI:** 10.1186/s12906-020-03092-2

**Published:** 2020-10-19

**Authors:** Huijin Lau, Suzana Shahar, Mazlyfarina Mohamad, Nor Fadilah Rajab, Hanis Mastura Yahya, Normah Che Din, Hamzaini Abdul Hamid

**Affiliations:** 1 Center for Healthy Aging and Wellness, Faculty of Health Sciences, Universiti Kebangsaan Malaysia, Jalan Raja Muda Abdul Aziz, 50300 Kuala Lumpur, Malaysia; 2 Centre for Diagnostic and Applied Sciences, Faculty of Health Sciences, Universiti Kebangsaan Malaysia, Jalan Raja Muda Abdul Aziz, 50300 Kuala Lumpur, Malaysia; 3 Center for Rehabilitation Sciences, Faculty of Health Sciences, Universiti Kebangsaan Malaysia, Jalan Raja Muda Abdul Aziz, 50300 Kuala Lumpur, Malaysia; 4 Department of Radiology, Faculty of Medicine, Universiti Kebangsaan Malaysia Medical Center, Jalan Yaacob Latif, Bandar Tun Razak, 56000 Batu 9 Cheras, Kuala Lumpur, Malaysia

**Keywords:** Brain-derived neurotrophic factor, fMRI, Medicinal plants, Mild cognitive impairment, Mood

## Abstract

**Background:**

*Persicaria minor* extract exhibits antioxidant and anti-inflammatory properties and has potential effects on cognitive function and mood. However, the effects of *P.minor* on brain activation and biomarkers have not been studied among older adults. This multicentre, randomized, double-blinded, placebo-controlled study aimed to investigate the effect of 6 months *P.minor* extract supplement (Biokesum®) on cognition, mood, biomarkers, and brain activation among older adults with Mild Cognitive Impairment (MCI).

**Method:**

A total of 36 Malaysian community-dwelling older adults with MCI (60–75-year-old) were randomized into Biokesum® (*n* = 18) and placebo group (n = 18). Each subject consumed one capsule of Biokesum® (250 mg/capsule) or placebo (maltodextrin, 280 mg/capsule) twice daily for 6 months. Cognitive function and mood were assessed at baseline, 3rd, and 6th-month using neuropsychological tests (MMSE, Digit Span, RAVLT, Digit Symbol, and Visual Reproduction) and Profile of Mood State (POMS) questionnaire. Blood lipid profile, fasting blood glucose, and biomarkers (MDA, LPO, COX-2, iNOS, and BDNF) were measured at baseline and 6th month. By the end of the intervention, there were 30 compliers (Biokesum®: *N* = 15; Placebo: N = 15) and 6 dropouts. For brain activation assessment, 15 subsamples (Biokesum®: *N* = 8; Placebo: *N* = 7) completed N-back and Stroop tasks during fMRI scanning at baseline and 6th month. The dorsolateral prefrontal cortex (Brodmann’s area 9 and 46) was identified as a region of interest (ROI) for brain activation analysis using SPM software.

**Results:**

Two-way mixed ANOVA analysis showed significant improvements in Visual Reproduction II (*p* = 0.012, partial η^2^ = 0.470), tension (*p* = 0.042, partial η^2^ = 0.147), anger (*p* = 0.010, partial η^2^ = 0.207), confusion (*p* = 0.041, partial η^2^ = 0.148), total negative subscales (*p* = 0.043, partial η^2^ = 0.145), BDNF (*p* = 0.020, partial η^2^ = 0.179) and triglyceride (*p* = 0.029, partial η^2^ = 0.237) following 6 months of Biokesum® supplementation. Preliminary finding also demonstrated significant improvement at 0-back task-induced right DLPFC activation (*p* = 0.028, partial η^2^ = 0.652) among subsamples in Biokesum® group. No adverse events were reported at the end of the study.

**Conclusion:**

Six months Biokesum® supplementation potentially improved visual memory, negative mood, BDNF, and triglyceride levels among older adults with MCI. Significant findings on brain activation at the right DPLFC must be considered as preliminary.

**Trial registration:**

Retrospectively registered on 30th August 2019 [ISRC TN12417552].

## Background

The rapid growth of the worldwide aging population has resulted in an increase in neurodegenerative disorders, such as Alzheimer’s disease, which results in cognitive function, physical functionality, and quality of life impairments [1]. Cognitive functions including memory, attention, processing speed, visuospatial perception, and problem solving deteriorate with age and are affected by both non-modifiable (age and genetics) and modifiable risk factors (lifestyle factors, hypertension, diabetes, and depression) [2–4]. Therefore, adopting a healthy lifestyle behavior is essential to promote cognitive and mental health [5, 6].

The presence of phenolic compounds and flavonoids in herbs and medicinal plants are being acknowledged for their potential benefits on human health [7–9]. Several recent studies have reported the beneficial effects of herbs or natural extract supplementation on cognitive performance, mood, biomarkers, and also brain activity. For example, the beneficial effects of coffee fruit extract [10, 11], *Ginkgo biloba* [12, 13], and curcumin [14, 15] on the brain-derived neurotrophic factor (BDNF), cognitive function and mood of older adults have been reported. Besides, consumptions of aloe polymannose multi nutrients complex, *Cistus incanus* herbal tea and chayote (*Sechium edule*) decreased oxidative stress marker such as malondialdehyde and lipoperoxides, inflammation markers tumor necrosis factor-alpha (TNF- α) and also triglyceride level among older adults [16–18]. Brain activity was also improved among healthy adults, AD and aMCI patients after the consumption of Chinese herbal decoctions [19, 20] and multi-ingredients herbal supplement that consists of *Bacopa monniera*, *Panax quinquefolius ginseng*, and whole coffee fruit extract [21].

*Persicaria minor* (*P.minor)* is an aromatic plant belongs to the family *Polygonaceae*, originating from Southeast Asian countries such as Indonesia, Vietnam, Thailand, and Malaysia [22]. *P.minor* leaf extract was found to have flavonoids such as quercetin, has been suggested to have antioxidant, anti-inflammation [23, 24], and antimicrobial properties [25]. To date, there were only two human studies investigated the effect of *P.minor* extract on cognitive function, mood, and quality of life. A study conducted by Udani [26] showed a significant improvement in cognitive function and mood after 3 weeks of SuperUlam supplementation. SuperUlam is a proprietary blend of natural ingredients including *P.minor* extract. Recent research using *P.minor* extract supplement (LineMinus™) also demonstrated a significant improvement in attention and memory, mood, and quality of life among middle-aged women after 6 weeks of supplementation [27]. The effects of *P.minor* have never been evaluated on neurotrophin, oxidative stress, and inflammation biomarkers and functional brain activity using functional magnetic resonance imaging (fMRI) especially among older adults with MCI.

To address this knowledge gap, a randomized, double-blind, placebo-controlled clinical trial was designed and conducted to evaluate the effects of 6 months *P.minor* extract supplement on cognitive function, mood, neurotrophin, oxidative stress, and inflammation biomarkers as well as fMRI brain activity among older adults with MCI.

## Methods

### Study design and ethical approval

This study was a multicentre, double-blinded, randomized, placebo-controlled trial with allocation ratio 1:1, which consists of three visits: (1) visit 1 (baseline), (2) visit 2 (3rd month) and (3) visit 3 (6th month). A total of 36 Malaysian community-dwelling older adults aged 60 to 75 with Mild Cognitive Impairment (MCI) were successfully recruited in this study. Out of 36 participants, 15 volunteered sub-samples participated in an fMRI assessment to evaluate the effect of *P.minor* extract supplement on brain activity (Biokesum®: *N* = 8; placebo: *N* = 7). The Medical Research Ethics Committee of Universiti Kebangsaan Malaysia (MRECUKM) (NN- 2017-036) approved the study protocol. Written informed consent was obtained from all participants before data collection. This study was also registered under the ISRCTN Registry (ISRCTN12417552) and conducted under Good Clinical Practice Guidelines and the ethical principles of the Declaration of Helsinki.

### Sample size calculation

With a two-sided 5% significance level and a power of 80%, the calculation for sample size is determined by using a Randomised Controlled Trials formula proposed by Zhong [28]. Regarding the study of Ma et al. [29], the mean difference of Digit Span score between treatment and control groups (3.4) with pooled standard deviation (10.95) were substituted into the formula. The calculated sample size was 15 per group.

### Study settings and participants

Participants were recruited from community-dwelling older adults’ population through poster advertising and invitation. Recruitment and data collection process were conducted at multiple senior citizen clubs located in Klang Valley, Malaysia from 11th March 2017 to 4th October 2017. Participants’ allocation was based on a simple randomization method using a computer-generated list of random numbers prepared by the investigator with no involvement in the clinical trial. All the study personnel and participants were kept blinded to the group assignment, study product distribution, and trial findings. The manufacturing company carried out the unblinding process after the data analysis was completed.

Potential participants were screened for eligibility based on inclusion and exclusion criteria in a health screening and one-to-one interview session before the start of the intervention. A participant information sheet was distributed to each participant during the screening session to provide relevant information on the clinical trial, which included the objective, study product, procedure, potential benefits, and risks, as well as the rights to refuse or withdraw. Older adults with MCI were determined based on the criteria published in the previous Malaysian population-based study of a neuroprotective model for healthy longevity using a series of neuropsychological tests [30]. Older adults with self-reported regular consumption of traditional herbs, vitamin or mineral supplements for the past 6 months, self-reported neurodegenerative diseases (i.e., dementia and Parkinson disease), depressive symptoms (score > 5 in Geriatric Depression Scale) or other serious medical conditions such as renal and kidney failure (based on blood analysis report) were excluded from the study.

**Fig. 1:**
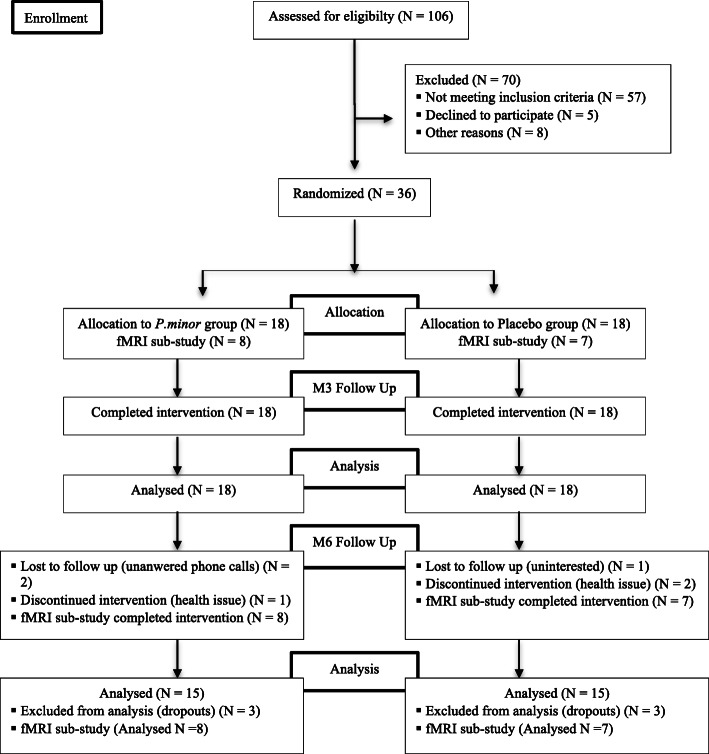
Consort Study Flow Chart

Figure 1 showed the consort study flow chart of the study. A total of 106 older adults age 60–75 years were enrolled and accessed for eligibility. Seventy of them were excluded from the study due to not meeting the inclusion criteria (*n* = 57), declined to participate (n = 5) and other personal reasons (*n* = 8). Participants who met the inclusion criteria were randomized into the placebo and Biokesum® group, with 18 subjects per arm. All the participants completed the study at baseline and the 3rd-month follow-up (*n* = 36). At the 6th month follow up, there were a total of 3 dropouts from each group due to uninterested to continue the study, lost to follow up, and health issue. In conclusion, the remaining 30 participants completed the 6 months of intervention and were included in the analysis. Per protocol population was used in the analysis of primary and secondary endpoints. All of the sub-samples (*N* = 15) also completed the fMRI assessment at the end of the intervention and were included in the analysis.

### Study product

Biokesum® is a patent-pending (P1 2,012,003,882) aqueous extract of *P. minor* supplement provided by the sponsor company Biotropics Malaysia Sdn Bhd. A finished product in the form of a capsule contains 250 mg of Biokesum® extract was developed and registered under the National Pharmaceutical Control Bureau (NPCB) with the registration number of MAL14015033T. Each capsule of Biokesum® contains bioactive compounds included quercetin-3-glucuronide (not less than 0.45%), quercitrin (not less than 0.15%), and total phenolic content (not less than 100 mg GAE/g dE). Toxicity, changes in behavior, and mortality were not observed in Winstar rats up to 2000 mg/kg doses in acute oral toxicity study [31].

The participants were randomized to received either *P.minor* extract supplement (Biokesum®) (250 mg/capsule) or sensory-identical placebo composed of maltodextrin (280 mg/capsule) administered by the researcher. The maltodextrin has been used as the major placebo substance generally and considered as Generally Regarded As Safe (GRAS) status [32, 33]. Previous study also suggested that maltodextrin is safe for human consumption with acute no observed adverse effect level (NOAEL) of 0.8 g/kg body weight for men and more than 1.0 g/kg body weight for women [34]. Although there was an animal study suggested that excessive ingestion of maltodextrin impaired spatial recognition memory and increased weight gain in Wistar rats [35], however, the amount of maltodextrin given to the animals were between 409 g to 447 g, which is much higher than the dose given in this study.

Both of the study products were manufactured in a Good Manufacturing Practice (GMP) facility. The participants were instructed to take the study products one capsule twice a day, in the morning and afternoon followed by a meal. Participants were reminded to take the study products through daily text messages and phone calls. Compliance of the subjects was monitored regularly by performing capsule count during follow-ups. The compliance rate of this study was 85.6%.

### Study procedure

The participants were determined for their physical health (blood pressure, pulse rate, height and body weight measurement), cognitive function and mood status at baseline (visit 1), visit 2 and visit 3. Dietary macronutrient and micronutrient intake of the participants was also assessed using the 7-days Diet History Questionnaire (DHQ) and adjusted as confounding factors in the statistical analysis. At baseline and visit 3, participants were requested to fast at least 10–12 h before the day of data collection for blood sampling purposes. Sub-samples who have volunteered to participate in neuroimaging study were also required to undergo an fMRI assessment at baseline and visit 3. The study products were distributed at baseline and visit 2. Adverse events were also recorded at the end of visits 2 and 3.

### Cognitive and mood assessments

The primary objective of this study was to determine the effect of *P. minor* extract supplement on cognitive function and mood state of older adults with MCI (Table 1). A series of cognitive tests (Mini-Mental State of Examination, Digit Span, Rey Auditory Verbal Learning Test, Digit Symbol, and Visual Reproduction) were used to assess global cognitive function, working and episodic memory, cognitive processing speed and visual memory of the participants. Their mood for the past 7 days was also accessed using the Profile of Mood State (POMS) questionnaire.

**Table 1:** Primary and secondary outcomes

Task	Cognitive domains and mood status assessments
**Primary Outcomes**	
MMSE	Global Cognitive function
Digit Span	Attention, short term, and working memory
RAVLT	Verbal immediate memory
VR I & II	Non-verbal memory and visuospatial function
Digit symbol substitution	Psychomotor speed
POMS	Tension, depression, anger, vigor, esteem related affect, fatigue and confusion
**Secondary Outcomes**	
Biomarkers	BDNF, Oxidative stress (LPO, MDA), Inflammatory markers (iNOS, COX2)
Biochemical blood profile	Lipid profile and blood glucose profile
fMRI	Brain activation at the dorsolateral prefrontal cortex

*MMSE* mini-mental state examination, *RAVLT* rey auditory verbal learning test, *VR* visual reproduction, *POMS* profile of mood state, *BDNF* brain-derived neurotrophic factor, *LPO* lipid hydroperoxides, *MDA* malondialdehyde, *iNOS* inducible nitric oxide synthase, *COX2* cyclooxygenase 2, *fMRI* functional magnetic resonance imaging

### Blood biochemical profile and biomarkers measurements

The secondary objective of this study was to determine the effect of the *P. minor* extract supplement on biomarkers and fMRI brain activation (Table 1). Biochemical profile and biomarkers measurement were carried out at baseline and visit 3. Participants were required to fast overnight for at least 10 h for blood sampling purposes conducted by a trained phlebotomist. The collected blood samples were stored in an icebox for the delivery purpose to a local laboratory for analysis. Biochemical blood profile included lipid profile, blood sugar profile, liver function test, and renal profile were analyzed at medical laboratory Quantum Diagnostic Sdn. Bhd., Selangor, Malaysia. The serum samples were stored under − 80 °C before biomarkers analysis. Determination of biomarkers included oxidative stress markers (malondialdehyde (MDA) and lipid hydroperoxide (LPO)), inflammation markers (inducible nitric oxide synthase (iNOS) and cyclooxygenase-2 (COX-2)) and brain-derived neurotrophic factor (BDNF) were carried out using commercial ELISA kits (Elabscience, Houston, Texas, USA) at Faculty of Health Sciences, Universiti Kebangsaan Malaysia, Kuala Lumpur, Malaysia.

### Neuroimaging study

Functional Magnetic Resonance Imaging (fMRI) is a non-invasive technique used to determine the brain activation areas. It measures the changes in blood oxygen level in the active areas of the brain through the Blood Oxygen Level Dependent (BOLD) fMRI technique. Eligible participants were invited to the Department of Radiology, Universiti Kebangsaan Malaysia Medical Centre (UKMMC), Cheras, Kuala Lumpur, Malaysia to undergo fMRI assessment conducted by a trained radiographer. Participants’ consents were obtained before the scan.

All of the sub-samples completed the anatomical scans lasting for 3 min before fMRI tasks. Participants were required to complete two visual memory tasks, i.e., N-back and Stroop Color-Word task, in a session during the scan. The task paradigms were presented using Superlab5 (Cedrus Corporation, San Pedro, California, USA). A brief description of the study procedure and memory tasks were given to the participants before the scan.

#### N-Back

**Fig. 2:**
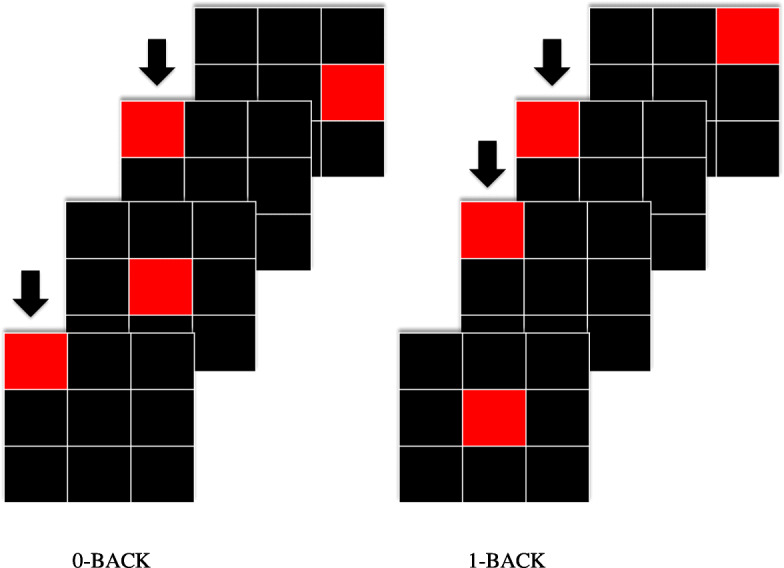
Schematic overview of N-back spatial task. The N-back spatial task consists of two paradigms: 0- and 1-back condition

N-back is widely used to access working memory. The task requires a cascade of cognitive processes included encoding and temporary storage of information, progressive updating incoming information. The N-back task with two paradigms: 0- and 1- back was used in this study to assess the working memory of older adults. Both 0- and 1- back consists of four task blocks (Fig. 2). A total of 15 trials for each block were displayed using a projection screen during each condition. In 0-back condition, participants need to identify the position of the target (red box) that is similar to the one as shown at the beginning of each block. In 1-back condition, participants need to identify the position of the target that is similar as the one preceding it. The stimulus was presented on a projection screen for 2000 milliseconds, with an inter-stimulus interval (ISI) of 30 s.

#### Stroop task

**Fig. 3:**
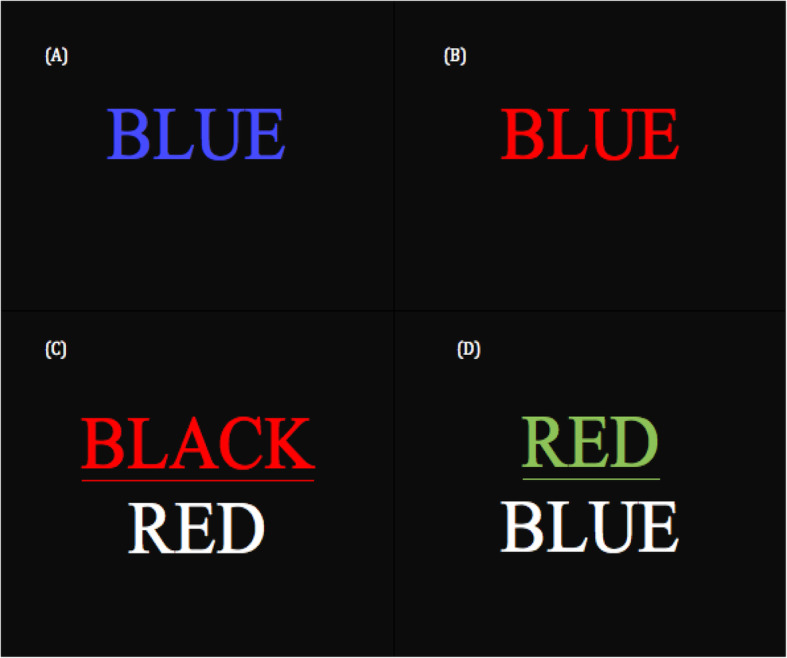
Schematic overview of Stroop task. (**a**) Matched color-word (**b**) Unmatched color-word (**c**) Color of the upper word matched with the meaning of the bottom word (**d**) Color of the upper word unmatched with the meaning of the bottom word

The Stroop task is a neuropsychological test used to assess the cognitive processing speed, flexibility, interference, and inhibition. In our study, the Stroop task consists of two paradigms. A total of four task blocks with 15 trials each was displayed in each paradigm. In the first paradigm, participants were presented with a series of color words (black, blue, yellow, green, red). These words appeared in different colors, sometimes matching the word (e.g., the word “blue”, displayed in blue ink), and sometimes not matching the word (i.e: the word “blue”, displayed in red ink). Participants were required to indicate the color in which the word is written, whether or not that matches the word itself. In the second paradigm, a white color word was displayed under a color word printed in different colors. The meaning of lower word sometimes is congruent with the color of the upper word (i.e: the lower word written as red, the upper word “black”, displayed in red ink) or sometimes is incongruent with the color of the upper word (i.e: the lower word written as red, the upper word “red”, displayed in green ink) (Fig. 3). Participants were required to indicate whether the color of the upper word corresponds with the meaning of the lower word or not. Participants shall not pay attention to the word but the color. Stimuli were presented on a projection screen for 2000 milliseconds, with an inter-stimulus interval of 30 s.

#### Image acquisition

A 3.0-T magnetic resonance scanner (MAGNETOM, Trio, Siemens, Erlagen, Germany) was used to acquire the fMRI data. T_1_-weighted image (repetition time (TR) = 1900 ms, echo time (TE) = 2.35 ms, voxel dimensions 1.0 × 1.0 × 1.0 mm, 250 × 250 voxels, 176 slices, slice thickness = 1 mm) and T2* weighted image during N-back and Stroop tasks (TR = 3000 ms, TE = 30 ms, 3 mm isotropic voxels, flip angle =90°, 27 slices, slice thickness = 4 mm) were obtained from the subjects. A total of 180 volumes were acquired for N-back and 170 volumes for the Stroop task. Results and raw images were then saved for further analysis using MATLAB (Mathworks Inc., Natick, MA, USA) and Statistical Parametric Mapping (SPM) software (SPM12; Wellcome Department of Cognitive Neurology, London, UK).

#### Pre-processing and functional imaging data analysis

Pre-processing and data analysis was performed using SPM software implemented in MATLAB. Prior to analysis, the functional images were realigned for motion correction and resliced to the mean image of the series. These functional images were then co-registered to the participants’s mean T1-weighted image, estimated against a standardized Montreal Neurological Institute (MNI) stereotaxic space. The spatial normalisation procedure utilized a 12-parameter affine transformation with a spatial transformation matrix. To reduce the intersubject variability, a spatial smoothing process with a 6-mm full-width half-maximum isotropic Gaussian kernel was conducted for all of the functional volumes.

Using the WFU PickAtlas library, the bilateral dorsolateral prefrontal cortex (DLPFC) (Brodmann’s area BA 9 and 46) was chosen as the region of interest (ROI). Percent signal change of the regions with peak activation (peak T-statistics) was extracted using the Marsbar tool, corrected for family-wise error (FWE), *p* < 0.05.

### Statistical analysis

All the data were analyzed using Statistical Package for Social Science (SPSS) version 22 (IBM, Armonk, New York, US). Independent *t*-test was employed to determine the differences in percentage mean change between the Biokesum® and the placebo group. The group by time effects were determined using two-way mixed ANOVA. Confounding factors such as age, caffeinated beverages intake, smoking status, educational level, baseline cognitive function (MMSE score), and nutrients intake (total calorie intake, vitamin A, C, thiamin, riboflavin, niacin, and iron) were controlled during the statistical analysis. Significant value was set at *p* < 0.05.

## Results

A total of 36 participants participated in this study at baseline. At the end of the intervention, a total of 3 participants from each study group were dropped from the study and leaving a total of 30 participants completed the study and data analysis. The dropouts were mainly due to loss of interest to continue the intervention. Other unidentified possible reasons could be health-related problems, personal problems, lack of time, or lack of motivation. As shown in Table 2, sociodemographic and self-reported health status were compared for both intervention and placebo groups. No significant differences were observed between the two groups at baseline. An additional file shows more details on baseline dietary nutrient intake (See Additional file 1). The findings of two-way mixed ANOVA intervention effects of the cognitive domains and mood assessments, blood biochemical profile and biomarkers, and fMRI brain activation were showed in the additional files.

**Table 2:** Comparison of baseline sociodemographic and self-reported health status between *P.minor* and placebo groups

	***P.minor*** (***N*** = 18)	Placebo (***N*** = 18)	Total (***N*** = 36)	***p*** value ^**†**^
Age^‡^	66.89 ± 4.00	65.94 ± 3.61	66.42 ± 0.63	0.462
Gender				
**Male**	3 (16.7)	5 (27.8)	8 (22.2)	0.423
**Female**	15 (83.3)	13 (72.2)	28 (77.8)	
Ethnicity				
**Malay**	16 (88.9)	17 (94.4)	33 (91.7)	0.220
**Chinese**	2 (11.1)	0 (9.1)	2 (5.6)	
**Indian**	0 (0)	1 (5.6)	1 (2.7)	
Educational Level				
**Not attending school**	1 (5.6)	3 (16.7)	4 (11.1)	0.293
**Primary school**	8 (44.4)	5 (27.8)	13 (36.1)	
**Secondary school**	9 (50.0)	7 (38.9)	16 (44.4)	
**Certificate/ Diploma**	0 (0)	2 (11.1)	2 (5.6)	
**Degree**	0 (0)	1 (5.6)	1 (2.8)	
Marital Status				
**Married**	9 (50.0)	13 (72.2)	22 (61.1)	0.305
**Widow/Widower**	9 (50.0)	5 (27.8)	14 (38.9)	
Smoking Status				
**Yes**	1 (5.6)	1 (4.5)	2 (5.6)	1.000
**No**	17 (94.4)	17 (94.4)	34 (94.4)	
Regular Supplement Consumer				
**Yes**	7 (38.9)	9 (50.0)	16 (44.4)	0.738
**No**	11 (61.1)	9 (50.0)	20 (55.6)	
Caffeinated Beverage Consumer				
**Yes**	17 (94.4)	17 (94.4)	34 (94.4)	1.000
**No**	1 (5.6)	1 (5.6)	2 (5.6)	
Working Status				
**Yes**	2 (11.1)	2 (11.1)	4 (11.1)	1.000
**No**	16 (88.9)	16 (88.9)	32 (88.9)	
Hypertension				
**Yes**	9 (50.0)	9 (50.0)	18 (50.0)	1.000
**No**	9 (50.0)	9 (50.0)	18 (50.0)	
Diabetes				
**Yes**	4 (22.2)	3 (16.7)	7 (19.4)	1.000
**No**	14 (72.8)	15 (83.3)	29 (80.6)	
Hyperlipidemia				
**Yes**	8 (44.4)	11 (61.1)	19 (52.8)	0.176
**No**	7 (38.9)	7 (28.9)	14 (38.9)	
**Not Sure**	3 (16.7)	0 (0.0)	3 (8.3)	

^**†**^ Crosstabs Chi-square test, not significant at *p* > 0.05

^‡^ Independent t-test, not significant at *p* > 0.05

### Cognitive domains and mood assessments

**Fig. 4:**
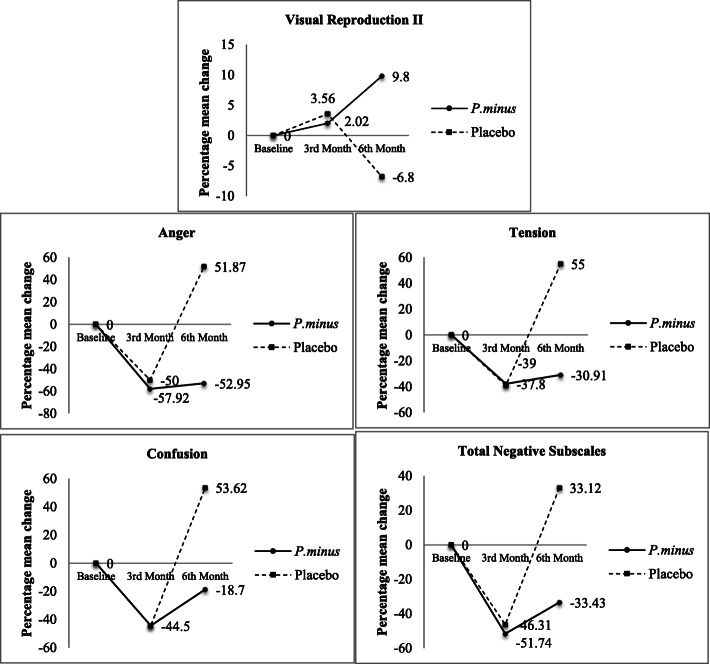
Percentage mean change of significant cognitive and mood parameters from baseline to 3rd-month and 6th-month follow-ups. After 6 months of intervention, percentage mean change of VRII was increased by 9.8% in the Biokesum® group and decreased by 6.8% in the placebo group. Percentage mean change of anger was decreased by 52.95% in the Biokesum® group, but increased by 51.87% in the placebo group. Percentage mean change of tension was decreased by 30.91% in the Biokesum® group, but increased by 55% in the placebo group. Percentage mean change of confusion was decreased by 44.5% in the Biokesum® group, but increased by 53.62% in the placebo group. Percentage mean change of total negative subscales was decreased by 33.43% in the Biokesum® group, but increased by 33.12% in the placebo group (Two-way mixed anova, *p* < 0.05)

Significant intervention effect was observed in Visual Reproduction II test (*p* = 0.012, partial η^2^ = 0.470) (See Additional file 1). The percentage mean difference of Visual Reproduction II score was increased following 6 months of intervention in the Biokesum® group (+ 9.80%) but decreased in the placebo group (− 6.80%) (Fig. 4). No significant intervention effects were observed in other cognitive parameters included Digit Span, RAVLT, and Digit Symbol (*p* > 0.05).

Significant intervention effect was also observed in mood states included tension (*p* = 0.042, partial η^2^ = 0.147), anger (*p* = 0.010, partial η^2^ = 0.207), confusion (*p* = 0.041, partial η^2^ = 0.148) and total negative subscales (*p* = 0.043, partial η^2^ = 0.145) (See Additional file 2). The percentage of mean differences was significantly reduced in tension (− 30.91%), anger (− 52.95%), confusion (− 18.7%) and total of negative subscales (− 33.43%) in the Biokesum® group but increased in placebo group by + 55%, + 51.87%, + 53.62%, + 33.12%, respectively at the end of the study (Fig. 4). No significant intervention effects were observed in other mood status included vigour, esteem related affect, total positive subscales and total mood disturbance (*p* > 0.05).

### Blood biochemical profile and biomarkers

**Fig. 5:**
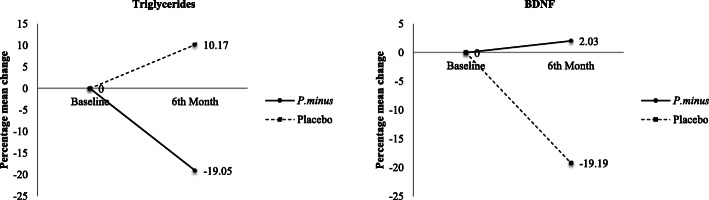
Percentage mean change of significant biomarkers from baseline to 3rd-month and 6th-month follow-ups. After 6 months of intervention, percentage mean change of triglycerides was decreased by 19.05% in the Biokesum® group, but increased by 10.17% in the placebo group. Percentage mean change of BDNF was increased by 2.03% in the Biokesum® group but decreased by 19.19% in the placebo group (Two-way mixed ANOVA, *p* < 0.05)

Significant intervention effect was observed in triglycerides (TG) concentration (*p* = 0.029, partial η^2^ = 0.537) (See Additional file 3). The TG percentage of mean difference was significantly reduced by 19.05% in the Biokesum® group but increased by 10.17% in the placebo group (Fig. 5). Brain-derived neurotrophic factor (BDNF) also demonstrated significant intervention effect (*p* = 0.020, partial η^2^ = 0.563) (See Additional file 2). The percentage mean difference was slightly increased by 2.03% in the Biokesum® group but decreased by 19.19% in the placebo group at the end of the intervention (Fig. 5). Other blood biochemical parameters included blood glucose profile, MDA, LPO, COX-2, and iNOS did not show any significant improvements following 6 months of intervention (*p* > 0.05).

### fMRI brain activation

**Fig. 6:**
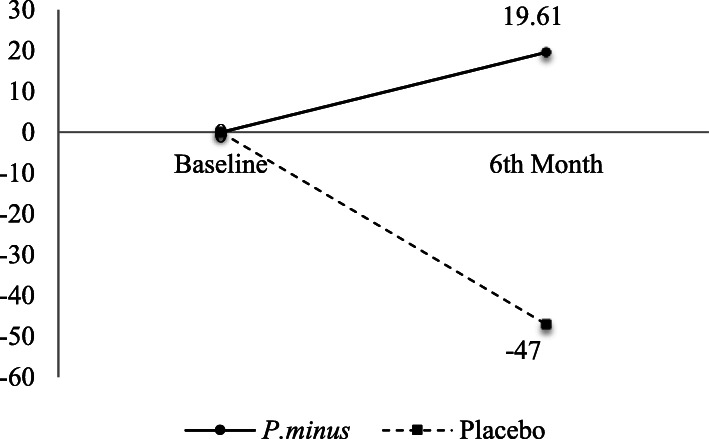
Percentage mean change of significant brain activation from baseline to 6th-month follow-ups. After 6 months of intervention, percentage mean change of the 0-back right DLPFC activation was increased by 19.61% and decreased by 47% in the placebo group (Two -way mixed ANOVA, *p* < 0.05)

0-back task-induced right DLPFC activation showed significant intervention effect (*p* = 0.028, partial η^2^ = 0.652) (See Additional file 4). The percentage mean difference of right DLPFC percent signal change was increased by 19.61% during the 0-back task in the Biokesum® group but decreased by 47% in the placebo group (Fig. 6). No significant intervention effects were observed in other parameters included 1-back and Stroop task-induced DLPFC activation (*p* > 0.05).

**Fig. 7:**
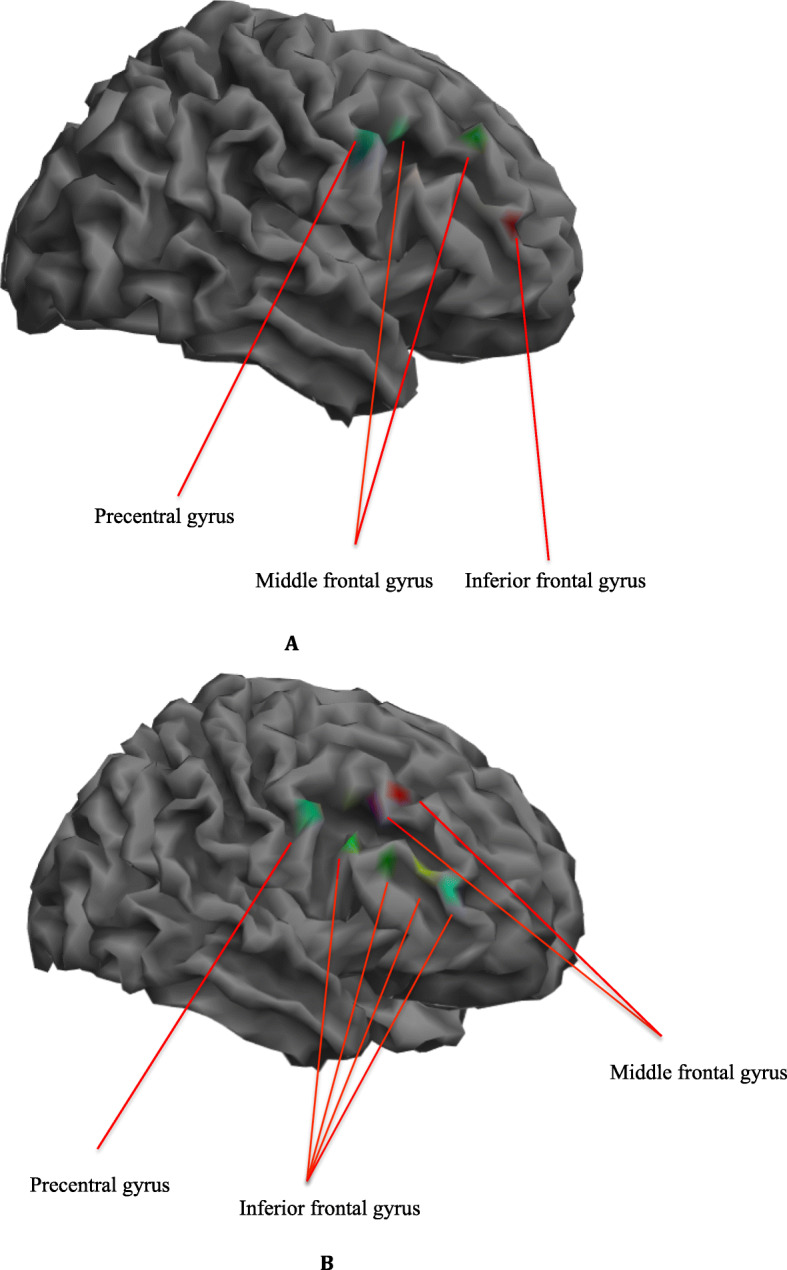
Right DLPFC activation during 0-back, 1-back and Stroop tasks on a volume rendered brain at baseline (**a**) and 6th month (**b**), family-wise error (FWE) corrected *p* < 0.05. More activation was observed during the brain tasks at the right DLPFC regions included precentral gyrus, inferior frontal gyrus, and middle frontal gyrus at 6th-month intervention when compared to baseline. (Blue area: 0-back task; Red area: 1-back task; Green area: Stroop task; Magenta area: 0-back and 1-back tasks; Cyan area: 0-back and Stroop tasks; Yellow area: 1-back and Stroop tasks)

Given this significant finding, the tasks-induced right DLPFC activation of the Biokesum® group were displayed on a volume rendered brain using ROI analysis at BA 9 and 46 (FWE, *p* < 0.05) (Fig. 7). As compared to baseline, more areas were activated included inferior frontal gyrus (IFG), middle frontal gyrus (MFG), and precentral gyrus (PrG) during the 0-back task following *P. minor* extract supplementation. IFG was the only region activated during the 1-back task at baseline, but activation was also observed at MFG after 6 months. Baseline activations were also observed at PrG and MFG during the Stroop task and at PrG and IFG after 6 months.

### Adverse events

No serious adverse events were reported in this study.

## Discussion

The primary finding of this research provided partial support for our hypothesis that 6 months *P. minor* extract (Biokesum®) supplement showed significant enhancement in visual memory but not other cognitive domains such as attention, verbal memory, and processing speed among older adults with MCI. Biokesum® supplement consists of phenolic compound quercetin 3-*O*-glucuronide (Q3G), a glucuronide conjugate of quercetin. It has been suggested that Q3G has a potential beneficial effect on cognitive health due to its ability in increasing protein secretion of BDNF, promoting neural stem cells migration and neurogenesis [36], inhibiting endoplasmic reticulum stress which contributed to oxidative stress and attenuating tau protein phosphorylation [37, 38].

A previous study using a similar *P. minor* supplement (previously known as LineMinus™) (500 mg/day) also reported significant improvements in Digit Span, Comprehensive Trail Making Test, and CNS vital signs in cognitive flexibility and executive function among healthy middle-aged women after 6 weeks of consumption [27]. Together with the current finding, both short and long duration consumption of *P. minor* extract supplement has the potential to improve cognitive domains such as attention, cognitive flexibility and executive function among middle-aged adults and visual memory among older adults with MCI. Nevertheless, we did not find any significant improvements in other cognitive domains. It could be due to the visual working memory impairment that emerged before the verbal working memory impairment among individuals with MCI as suggested by Emrani et al. [39]. Therefore, the effect of the *P. minor* extract supplement may be more notable in the visual memory domain as compared to other cognitive domains in this study.

Significant improvements were observed in most of the negative mood status particularly tension, anger, confusion, and total negative subscales in the present study. Our findings were in line with the previous study [27], which also demonstrated significant improvements in tension, depression, anger, and total mood disturbance among healthy middle-aged women with mood disturbance. Quercetin and Q3G have been suggested to have an anti-depression ability [40]. These flavonoids were reported to have an impact on gamma-aminobutyric acid (GABA) receptors producing sedation, anxiolytic, or anticonvulsive effects [41]*.* Also, quercetin can inhibit monoamine oxidase (MAO), which involved in mood disorder, depression, and oxidative stress [42, 43]. This can be supported by the recent animal study, which reported that 14 days of ingestion of quercetin (20 mg/kg) could reverse stress-induced anxiety and depression in mice [44]. Another study by Metha et al. [45] demonstrated that mice ingested with quercetin (30 mg/kg, 21 days) reversed the effects of chronic unpredicted stress on depression and anxiety. Therefore, the presence of the flavonoid in the supplement might explain its beneficial effects in reducing negative mood among older adults with MCI.

To our knowledge, this is also the first study to examine the effect of the *P. minor* extract supplement on biomarkers. Our findings revealed that the BDNF concentration was significantly higher in the Biokesum® group as compared to the placebo group following 6 months of intervention. Previous animal studies [36, 46] highlighted that quercetin and Q3G significantly increased the mRNA expression and secretion of BDNF in rats, which contribute to the neural stem cell proliferation. The possible underlying mechanism could be the quercetin activates BDNF-TrkB and its associated signaling pathway, which eventually, results in phosphorylation of CREB, followed by an increase in extracellular signal-regulated kinase (ERK) and BDNF [47, 48]. Therefore, quercetin interactions with these pathways may be responsible for its role in the central nervous system.

We also postulate that the improvement in the BDNF level may have an indirect effect on the mood findings reported in the present study. Reduction in BDNF levels has been reported in persons with depression [49]. Increased anxiety-related behaviors, decreased ventromedial prefrontal cortex volume and memory deficits have been observed in mice with BDNF Val66Met polymorphism [50, 51]. These findings suggested that the BDNF Val66Met polymorphism might cause smaller prefrontal cortex development and increased the risk of cognitive decline and mood disorders. Nevertheless, minimal increment in the BDNF level has been noted in this study and we are aware that the significant result could be possibly due to the greater reduction in the level as seen in the placebo group.

Triglyceride concentration was also significantly reduced following 6 months of *P. minor* extract supplementation. This finding was consistent with Yahya et al. [27], which also reported a significant reduction in lipid profile, particularly total cholesterol to HDL ratio. More than half of the subjects in this study were reported to have hyperlipidemia (52.8%). Hyperlipidemia may induce the production of the reactive oxygen species (ROS) from the mitochondrial electron system lead to the generation of lipid peroxide radicals and lipid peroxidation. Increased lipid peroxidation is thought to be a consequence of oxidative stress and it is associated with Alzheimer’s disease [52]. Our findings can be supported by the preclinical study, which suggested that *P. minor* extract possessed an antihyperlipidemic effect by significantly reduced lipid levels (total cholesterol, TG, and LDL) in a rat model [53]. It has been suggested it could be due to the flavonoids myricetin and quercetin that present in the *P. minor* extract. A recent animal study showed that high-fat induced obese rat that consumed a quercetin-rich formulation supplement presented a much less lipid accumulation and smaller size of adipocytes [54]. A study by Seo et al. [55] has been suggested that quercetin inhibits lipid accumulation and obesity-induced inflammation in both cell and animal models. Mitogen-activated protein kinase (MAPK) signaling factors played a role in adipogenesis and inflammation and that adipokines MCP-1 and TNF-α increased the recruitment of macrophages in adipose tissue. Quercetin was found to have the ability in suppressing the MAPK signaling factors (extracellular signal-regulated kinases 1 and 2 (ERK1/2), JNK, p38MAPK, MCP-1, and TNF-α) that played a role in adipogenesis and inflammation. The findings also revealed that quercetin inhibits the secretion of the inflammatory cytokines IL-1β and IL-6 and stimulates the secretion of the anti-inflammatory cytokine, IL-10 [55]. Therefore, quercetin may play a role in reducing the TG level in the present study. Other than its antihyperlipidemic effect, quercetin was reported to possess antidiabetic effect by inhibiting intestinal glucose absorption, increasing insulin secretion, improved glucose utilization in peripheral tissue through increased protein expression and phosphorylation of insulin receptor [56]. However, we did not find any significant findings on the blood glucose profile. The lack of observed effects of the *P. minor* extract supplement maybe because the present study was not focused on the subjects with T2DM. It also remains possible that the effect of herbal supplementation on the biochemical profile can vary according to dosage, duration, and the forms of supplementation given to the subjects.

The previous in vitro and animal studies that demonstrated strong antioxidant and anti-inflammation influences of *P. minor* extract did not support our findings on antioxidant and anti-inflammation biomarkers. George et al. [57] highlighted that the ethanolic *P. minor* extract significantly inhibited cyclooxygenase-1 (COX-1) and lipooxygenase (5-LOX) activities, however, the inhibitory effect on cyclooxygenase-2 (COX-2) was minimal. The same research also reported that aqueous *P. minor* extract (100 and 300 mg/kg) significantly reduced inflammation of carrageenan-induced rat paw edema. Despite promising results in in-vitro and animal studies, the results of the present study raise questions about the generalizability of these findings to human populations. One of the possible reasons could be the interindividual variation in quercetin bioavailability reduced the antioxidant and anti-inflammation capacity [58, 59]. It remains plausible that the significant reduction in lipid levels might suppress lipid peroxidation, which in turn contributes to the insignificant findings on oxidative stress and inflammation markers.

The effect of *P. minor* extract supplement on fMRI brain activation was examined among subsamples in the present study. A significant increase in the activation was observed at the right DLPFC during the 0-back task following 6 months of supplementation. Activation at the right DLPFC has been suggested to be more dominant in visuospatial working memory [33, 60]. According to Barbey et al. [61], right DLPFC is responsible for manipulating information beyond working memory in a wider range of reasoning contexts, such as arithmetic and spatial reasoning, goal-directed behavior, and decision-making. These cognitive skills are important for older adults to maintain their quality of life. A previous animal study reported that flavonoid quercetin could protect mice against a reduction in cerebral blood flow and memory impairment [62]. Quercetin also possessed the ability to protect human brain microvascular endothelial cells from toxicity induced by fibrillar amyloid-β, a signature hallmark of Alzheimer’s disease [63]. Progressive accumulation of amyloid-β in and around blood vessels in the brain disrupted Blood-Brain Barrier (BBB) permeability, which in turn reduces cerebral blood flow to the brain [64]. Sufficiency of cerebral blood flow is important to maintain brain function, and insufficiency causes neurodegeneration [65].

Activation at right DLPFC, particularly right precentral gyrus, middle frontal gyrus, and inferior frontal gyrus were observed in the present study. Similarly, in a study conducted by Kwon et al. [66], the subjects also demonstrated more activation in the right hemisphere such as middle frontal gyrus and inferior frontal gyrus during visuospatial N-back task. Middle frontal gyrus (MFG) is associated with age-related decline in episodic memory retrieval. It can retrieve information about previously experienced events or items in rich contextually details [67]. Right middle frontal gyrus specifically activated during learning of non-verbal information, for example patterns. While left MFG activated in response to literary than numeracy [68]. Right precentral gyrus has also been implicated in response inhibition [69]. Therefore, it is not surprising that these regions showed more activation during visual brain tasks.

The strengths and limitations of the current study must be considered. The primary strength of the current study was the use of a randomized, double-blinded placebo-controlled method to access the efficacy of *P.minor* extract supplement on various aspects from neuropsychological assessments to blood biomarkers and neuroimaging for 6 months. Furthermore, this study was focused on older adults with mild cognitive impairment who are at high risk of getting Alzheimer’s disease. In addition, the use of neuroimaging technique is another strength of this study. An fMRI is a noninvasive imaging tool and there is no exposure to ionizing radiation. Therefore, it is suitable to be used in the studies involving older adults. Besides, it can also provide an insight into the underlying cerebral hemodynamic response to herbal supplementation. Thus, fMRI is more reliable and sensitive as compared to objective cognitive assessments when determining the cerebral response changes among those with risk of cognitive impairment [70]. It is also important to note some limitations of the present study. First, the functional evidence was limited to small sample size. Therefore, the present functional finding must be considered as preliminary. Besides, we used self-report to evaluate participants’ mood and this may lead to recall and selective bias. Future studies would benefit from using a larger and more diverse sample size to confirm the neuroprotective and mood regulation effects of *P.minor* extract supplementation.

## Conclusions

The findings from the current research suggested that 6 months *of P.minor* extract (Biokesum®) supplementation can significantly improve visuospatial memory, tension, anger, confusion, total negative subscales, triglyceride and BDNF level among older adults with MCI. Significant finding on the right DLPFC activation was based on subsamples only. Therefore, the results must be considered preliminary until such effects can be studied further in a larger sample size. These findings may serve as a knowledge base for other herbal or natural extracts supplementation studies in the future.

## Supplementary information

**Additional file 1.** Baseline dietary nutrients intake.

**Additional file 2.** Intervention effect of neurocognitive assessments and POMS.

**Additional file 3.** Intervention effect of blood biochemical profile and biomarkers.

**Additional file 4.** Intervention effect of fMRI brain activation (percent signal change).

## Data Availability

The datasets used and/or analysed during the current study available from the corresponding author on reasonable request.

## Abbreviations

ALT: Alanine transferase
BDNF: Brain-derived neurotrophic factor
BOLD: Blood oxygen level dependent
COX2: Cyclooxygenase 2
DHQ: Diet history questionnaire
DLPFC: Dorsolateral prefrontal cortex
ELISA: Enzyme-linked immunosorbent assay
FBC: Full blood count
FBG: Fasting blood glucose
fMRI: Functional magnetic resonance imaging
GMP: Good manufacturing practice
HbA1c: Glycated hemoglobin A1c
HOMA: Homeostatic model assessment
iNOS: Inducible nitric oxide synthase
LP: Lipid profile
LFT: Liver function test
MCI: Mild cognitive impairment
MDA: Malondialdehyde
MFG: Middle frontal gyrus
MMSE: Mini-mental state of examination
NPCB: National Pharmaceutical Control Bureau
*P. minor*: *Persicaria minor*
POMS: Profile of mood state
RAVLT: Rey auditory verbal learning test
TG: Triglyceride
VR: Visual reproduction
