# Supplementary material for: The effects of six months Persicaria minor extract supplement among older adults with mild cognitive impairment: a double-blinded, randomized, and placebo-controlled trial
Source: BMC Complement Med Ther. 2020 Oct 19;20:315. doi: 10.1186/s12906-020-03092-2 (PMC7574246; doi:10.1186/s12906-020-03092-2)
Supplement: Supplementary file 1 — Additional file 1. Baseline dietary nutrients intake. [file 12906_2020_3092_MOESM1_ESM.docx]

**Additional File 1 Baseline dietary nutrients intake**

| **Dietary Nutrients Intake** | **Total (n = 36)** | **Normal Range** |
| --- | --- | --- |
| Energy (kcal) | 1530.21 ± 391.24 | 1770-2330 |
| Protein (g) | 55.90 ± 17.44 | 50-58 |
| Carbohydrate (%) | 46.99 ± 2.79 | 50-65 |
| Fat (g) | 66.31 ± 24.60 | 49-68 |
| Total fibre (g) | 5.15 ± 2.28 | 20-30 |
| Vitamin A (RE) | 734.39 ± 285.00 | 600 |
| Vitamin C (mg) | 83.48 ± 43.31 | 70 |
| Vitamin E (mg) | 3.90 ± 1.77 | 7.5-10 |
| Thiamin (mg) | 0.58 ± 0.22 | 1.1-1.2 |
| Riboflavin (m) | 0.92 ± 0.53 | 1.1-1.3 |
| Niacin (mg) | 8.94 ± 3.38 | 14-16 |
| Vitamin B6 (mg) | 0.94 ± 0.53 | 1.5-1.7 |
| Folate (μg) | 122.30 ± 111.62 | 400 |
| Vitamin B12 (μg) | 3.68 ± 2.98 | 4 |
| Sodium (mg) | 2409.60 ± 633.97 | 2300 |
| Potassium (mg) | 1488.13 ± 551.34 | 4700 |
| Calcium (mg) | 421.66 ± 400.04 | 1000-1200 |
| Iron (mg) | 11.11 ± 4.39 | 11-14 |
| Phosphorus (mg) | 722.68 ± 235.19 | 4000 |
| Magnesium (mg) | 120.96 ± 52.23 | 420 |
| Zinc (mg) | 2.72 ± 1.24 | 4.3-6.3 |
| Copper (mg) | 0.48 ± 0.21 | 0.9 |
| Manganese (mg) | 0.64 ± 1.48 | 1.8 |
| Selenium (μg) | 26.08 ± 16.54 | 23-31 |
| Chromium (μg) | 0.0001 ± 0.02 | 20-30 |
| Molybdenum (mg) | 0.06 ± 2.24 | 0.045 |
