# Supplementary material for: The effects of six months Persicaria minor extract supplement among older adults with mild cognitive impairment: a double-blinded, randomized, and placebo-controlled trial
Source: BMC Complement Med Ther. 2020 Oct 19;20:315. doi: 10.1186/s12906-020-03092-2 (PMC7574246; doi:10.1186/s12906-020-03092-2)
Supplement: Supplementary file 2 — Additional file 2. Intervention effect of neurocognitive assessments and POMS. [file 12906_2020_3092_MOESM2_ESM.docx]

**Additional file 2 - Intervention effect of neurocognitive assessments and POMS**

|  | *P.minor*  (N = 15) | Placebo  (N = 15) | Group x Time Effect | |
| --- | --- | --- | --- | --- |
|  |  |  | *p* | Partial Eta Squared |
| **NEUROCOGNITIVE ASSESSMENTS** | | | | |
| **Digit Span** |  |  | **0.614** | **0.067** |
| *Baseline* | 13.53 ± 2.39 | 12.47 ± 3.18 |  |  |
| *3^rd^ month* | 14.93 ± 3.62 | 14.60 ± 3.42 |  |  |
| *6^th^ month* | 14.07 ± 3.56 | 14.00 ± 3.53 |  |  |
| **RAVLT Immediate Recall** | |  | **0.912** | **0.013** |
| *Baseline* | 6.80 ± 1.61 | 7.33 ± 2.92 |  |  |
| *3^rd^ month* | 9.40 ± 2.53 | 8.93 ± 3.49 |  |  |
| *6^th^ month* | 8.80 ± 2.04 | 9.40 ± 2.67 |  |  |
| **RAVLT Delayed Recall** | |  | **0.053** | **0.343** |
| *Baseline* | 4.67 ± 2.64 | 7.00 ± 2.73 |  |  |
| *3^rd^ month* | 8.33 ± 3.44 | 8.67 ± 4.07 |  |  |
| *6^th^ month* | 7.40 ± 3.96 | 9.40 ± 2.95 |  |  |
| **Digit Symbol** |  |  | **0.073** | **0.312** |
| *Baseline* | 31.87 ± 12.71 | 36.87 ± 16.61 |  |  |
| *3^rd^ month* | 33.93 ± 13.08 | 38.20 ± 19.90 |  |  |
| *6^th^ month* | 33.40 ± 16.27 | 42.20 ± 14.84 |  |  |
| **Visual Reproduction Immediate Recall** | | | **0.855** | **0.022** |
| *Baseline* | 28.33 ± 7.74 | 32.13 ± 6.48 |  |  |
| *3^rd^ month* | 29.73 ± 7.04 | 33.40 ± 6.63 |  |  |
| *6^th^ month* | 26.67 ± 9.48 | 32.60 ± 8.18 |  |  |
| **Visual Reproduction Delayed Recall** | | | **0.012^*^** | **0.470** |
| *Baseline* | 27.87 ± 8.61 | 31.47 ± 8.94 |  |  |
| *3^rd^ month* | 26.67 ± 9.48 | 32.60 ± 8.18 |  |  |
| *6^th^ month* | 30.60 ± 8.12 | 29.33 ± 10.31 |  |  |
|  |  |  |  |  |
| **POMS** |  |  |  |  |
| **Tension** |  |  | **0.042^*^** | **0.147** |
| *Baseline* | 4.53 ± 4.97 | 4.00 ± 3.57 |  |  |
| *3^rd^ month* | 3.20 ± 2.78 | 2.67 ± 2.13 |  |  |
| *6^th^ month* | 3.13 ± 2.90 | 6.20 ± 3.90 |  |  |
| **Angry** |  |  | **0.010^*^** | **0.207** |
| *Baseline* | 4.40 ± 3.66 | 3.47 ± 2.59 |  |  |
| *3^rd^ month* | 2.00 ± 2.48 | 2.00 ± 2.42 |  |  |
| *6^th^ month* | 2.07 ± 2.25 | 5.27 ± 4.23 |  |  |
| **Fatigue** |  |  | **0.482** | **0.051** |
| *Baseline* | 4.73 ± 4.74 | 5.00 ± 4.19 |  |  |
| *3^rd^ month* | 1.53 ± 2.10 | 3.40 ± 3.74 |  |  |
| *6^th^ month* | 3.67 ± 3.44 | 5.00 ± 3.87 |  |  |
| **Depression** |  |  | **0.372** | **0.068** |
| *Baseline* | 3.53 ± 5.13 | 3.73 ± 3.08 |  |  |
| *3^rd^ month* | 1.73 ± 2.31 | 1.33 ± 1.45 |  |  |
| *6^th^ month* | 2.07 ± 2.49 | 4.33 ± 3.52 |  |  |
| **Esteem Related Affect** | |  | **0.166** | **0.120** |
| *Baseline* | 13.60 ± 2.10 | 13.67 ± 2.99 |  |  |
| *3^rd^ month* | 13.87 ± 1.89 | 13.47 ± 3.04 |  |  |
| *6^th^ month* | 14.33 ± 2.69 | 15.20 ± 3.99 |  |  |
| **Vigour** |  |  | **0.115** | **0.143** |
| *Baseline* | 9.73 ± 3.26 | 10.80 ± 4.09 |  |  |
| *3^rd^ month* | 9.67 ± 2.06 | 9.00 ± 3.87 |  |  |
| *6^th^ month* | 8.73 ± 3.08 | 11.40 ± 5.14 |  |  |
| **Confusion** |  |  | **0.041^*^** | **0.148** |
| *Baseline* | 3.53 ± 3.46 | 3.73 ± 3.11 |  |  |
| *3^rd^ month* | 2.07 ± 2.76 | 2.33 ± 1.54 |  |  |
| *6^th^ month* | 2.87 ± 3.02 | 5.73 ± 3.35 |  |  |
| **Total Negative Subscales** | |  | **0.043^*^** | **0.145** |
| *Baseline* | 20.73 ± 18.99 | 19.93 ± 14.10 |  |  |
| *3^rd^ month* | 10.53 ± 11.08 | 11.73 ± 8.90 |  |  |
| *6^th^ month* | 13.80 ± 12.01 | 26.53 ± 15.67 |  |  |
| **Total Positive Subscales** | |  | **0.162** | **0.122** |
| *Baseline* | 23.33 ± 4.22 | 24.47 ± 6.55 |  |  |
| *3^rd^ month* | 23.53 ± 3.52 | 22.47 ± 6.46 |  |  |
| *6^th^ month* | 23.60 ± 5.14 | 26.80 ± 8.39 |  |  |
| **Total Mood Disturbance** | |  | **0.228** | **0.100** |
| *Baseline* | 97.40 ± 19.91 | 95.47 ± 18.57 |  |  |
| *3^rd^ month* | 87.00 ± 12.63 | 89.27 ± 13.44 |  |  |
| *6^th^ month* | 90.20 ± 14.64 | 99.73 ± 19.65 |  |  |

* Significant at *p* < 0.05
