# Supplementary material for: The effects of six months Persicaria minor extract supplement among older adults with mild cognitive impairment: a double-blinded, randomized, and placebo-controlled trial
Source: BMC Complement Med Ther. 2020 Oct 19;20:315. doi: 10.1186/s12906-020-03092-2 (PMC7574246; doi:10.1186/s12906-020-03092-2)
Supplement: Supplementary file 3 — Additional file 3. Intervention effect of blood biochemical profile and biomarkers. [file 12906_2020_3092_MOESM3_ESM.docx]

**Additional file 3 - Intervention effect of blood biochemical profile and biomarkers**

|  | *P.minor*  (N = 15) | Placebo  (N = 15) | Group x Time Effect | |
| --- | --- | --- | --- | --- |
|  |  |  | *p* | Partial Eta Squared |
| **TCH (mmol/L)** |  |  | **0.976** | **0.000** |
| *Baseline* | 5.37 ± 0.86 | 5.14 ± 0.84 |  |  |
| *6^th^ month* | 5.43 ± 0.65 | 5.28 ± 0.76 |  |  |
| **TG (mmol/L)** |  |  | **0.029^*^** | **0.237** |
| *Baseline* | 1.47 ± 0.60 | 1.18 ± 0.49 |  |  |
| *6^th^ month* | 1.19 ± 0.41 | 1.30 ± 0.41 |  |  |
| **HDL (mmol/L)** |  |  | **0.810** | **0.003** |
| *Baseline* | 1.46 ± 0.26 | 1.56 ± 0.42 |  |  |
| *6^th^ month* | 1.47 ± 0.24 | 1.58 ± 0.48 |  |  |
| **LDL (mmol/L)** |  |  | **0.580** | **0.016** |
| *Baseline* | 3.25 ± 0.91 | 3.05 ± 1.01 |  |  |
| *6^th^ month* | 3.34 ± 0.61 | 3.06 ± 0.85 |  |  |
| **TCH: HDL Ratio** |  |  | **0.695** | **0.008** |
| *Baseline* | 3.81 ± 0.79 | 3.52 ± 1.12 |  |  |
| *6^th^ month* | 3.73 ± 0.67 | 3.57 ± 1.05 |  |  |
| **HbA1C %** |  |  | **0.362** | **0.044** |
| *Baseline* | 5.83 ± 0.80 | 5.77 ± 1.08 |  |  |
| *6^th^ month* | 5.87 ± 0.88 | 5.85 ± 1.42 |  |  |
| **Glucose (mmol/L)** |  |  | **0.284** | **0.060** |
| *Baseline* | 5.49 ± 1.22 | 5.51 ± 1.45 |  |  |
| *6^th^ month* | 5.61 ± 1.65 | 5.99 ± 3.57 |  |  |
| **Insulin (uIU/mL)** |  |  | **0.222** | **0.077** |
| *Baseline* | 17.11 ± 17.44 | 7.93 ± 3.49 |  |  |
| *6^th^ month* | 10.37 ± 6.99 | 6.17 ± 3.92 |  |  |
| **HOMA Bcell Function %** |  |  | **0.848** | **0.002** |
| *Baseline* | 178.00 ± 135.52 | 92.87 ± 37,64 |  |  |
| *6^th^ month* | 131.87 ± 124.03 | 73.13 ± 45.75 |  |  |
| **HOMA IR** |  |  | **0.244** | **0.071** |
| *Baseline* | 4.39 ± 5.51 | 2.07 ± 1.41 |  |  |
| *6^th^ month* | 2.76 ± 2.34 | 1.92 ± 2.45 |  |  |
| **Sodium (mmol/L)** |  |  | **0.309** | **0.054** |
| *Baseline* | 143.47 ± 1.68 | 144.00 ± 1.51 |  |  |
| *6^th^ month* | 143.60 ± 1.24 | 133.93 ± 37.11 |  |  |
| **Potassium (mmol/L)** |  |  | **0.590** | **0.016** |
| *Baseline* | 4.06 ± 0.45 | 3.96 ± 0.50 |  |  |
| *6^th^ month* | 3.96 ± 0.27 | 3.75 ± 1.13 |  |  |
| **Chloride (mmol/L)** |  |  | **0.365** | **0.043** |
| *Baseline* | 102.67 ± 1.39 | 102.20 ± 1.74 |  |  |
| *6^th^ month* | 101.33 ± 2.22 | 94.27 ± 26.13 |  |  |
| **Urea (mmol/L)** |  |  | **0.328** | **0.050** |
| *Baseline* | 3.51 ± 1.12 | 4.13 ± 0.87 |  |  |
| *6^th^ month* | 3.63 ± 1.06 | 3.93 ± 1.57 |  |  |
| **Uric (umol/L)** |  |  | **0.559** | **0.018** |
| *Baseline* | 324.33 ± 136.92 | 317.67 ± 54.89 |  |  |
| *6^th^ month* | 321.80 ± 138.68 | 291.40 ± 100.59 |  |  |
| **Creatinine (umol/L)** |  |  | **0.198** | **0.086** |
| *Baseline* | 62.33 ± 13.72 | 72.80 ± 24.82 |  |  |
| *6^th^ month* | 64.00 ± 17.53 | 75.27 ± 26.78 |  |  |
| **eGFR ml/min173m^2^** |  |  | **0.109** | **0.129** |
| *Baseline* | 87.67 ± 11.93 | 82.60 ± 18.90 |  |  |
| *6^th^ month* | 86.60 ± 13.14 | 81.47 ± 19.20 |  |  |
| **Total Protein (g/L)** |  |  | **0.793** | **0.004** |
| *Baseline* | 72.33 ± 3.90 | 70.93 ± 3.97 |  |  |
| *6^th^ month* | 73.67 ± 3.66 | 71.13 ± 3.66 |  |  |
| **Albumin (g/L)** |  |  | **0.379** | **0.041** |
| *Baseline* | 43.33 ± 2.06 | 44.40 ± 1.92 |  |  |
| *6^th^ month* | 43.73 ± 2.09 | 43.93 ± 1.83 |  |  |
| **Globulin (g/L)** |  |  | **0.777** | **0.004** |
| *Baseline* | 29.00 ± 3.55 | 26.53 ± 3.31 |  |  |
| *6^th^ month* | 29.93 ± 3.20 | 27.20 ± 2.98 |  |  |
| **Biomarkers** |  |  |  |  |
| **MDA** |  |  | **0.492** | **0.023** |
| *Baseline* | 176.59 ± 24.20 | 176.31 ± 25.32 |  |  |
| *6^th^ month* | 163.49 ± 24.95 | 172.27 ± 35.93 |  |  |
| **iNOS** |  |  | **0.970** | **0.000** |
| *Baseline* | 63.74 ± 118.89 | 31.48 ± 33.40 |  |  |
| *6^th^ month* | 31.19 ± 14.62 | 23.94 ± 10.26 |  |  |
| **COX-2** |  |  | **0.249** | **0.063** |
| *Baseline* | 1.96 ± 0.35 | 1.89 ± 0.27 |  |  |
| *6^th^ month* | 1.86 ± 0.34 | 1.73 ± 0.57 |  |  |
| **BDNF** |  |  | **0.020^*^** | **0.179** |
| *Baseline* | 77.22 ± 34.68 | 66.95 ± 39.71 |  |  |
| *6^th^ month* | 78.79 ± 33.84 | 54.10 ± 24.05 |  |  |

* Significant at *p* < 0.05

*** Significant at *p* < 0.001
