# Supplementary material for: The effects of six months Persicaria minor extract supplement among older adults with mild cognitive impairment: a double-blinded, randomized, and placebo-controlled trial
Source: BMC Complement Med Ther. 2020 Oct 19;20:315. doi: 10.1186/s12906-020-03092-2 (PMC7574246; doi:10.1186/s12906-020-03092-2)
Supplement: Supplementary file 4 — Additional file 4. Intervention effect of fMRI brain activation (percent signal change). [file 12906_2020_3092_MOESM4_ESM.docx]

**Additional file 4 - Intervention effect of fMRI brain activation (percent signal change)**

|  | *P.minor*  (N = 8) | Placebo  (N = 7) | Group x Time Effect | |
| --- | --- | --- | --- | --- |
|  |  |  | *p* | Partial Eta Squared |
| **N-BACK TASKS** | | | | |
| **0-Back Left DLPFC Activation** | | | **0.666** | **0.066** |
| *Baseline* | 1.00 ± 0.83 | 0.88 ± 0.43 |  |  |
| *6^th^ month* | 1.15 ± 1.12 | 0.50 ± 0.40 |  |  |
| **0-Back Right DLPFC Activation** | | | **0.028*** | **0.652** |
| *Baseline* | 1.02 ± 0.43 | 1.00 ± 0.57 |  |  |
| *6^th^ month* | 1.22 ± 0.60 | 0.53 ± 0.43 |  |  |
| **1-Back Left DLPFC Activation** | | | **0.248** | **0.188** |
| *Baseline* | 1.21 ± 0.67 | 0.66 ± 0.42 |  |  |
| *6^th^ month* | 0.94 ± 0.54 | 0.72 ± 0.46 |  |  |
| **1-Back Right DLPFC Activation** | | | **0.676** | **0.065** |
| *Baseline* | 1.11 ± 0.44 | 0.98 ± 0.54 |  |  |
| *6^th^ month* | 1.36 ± 1.17 | 0.52 ± 0.40 |  |  |
| **STROOP TASK** | | | |  |
| **Left DLPFC Activation** | | | **0.729** | **0.061** |
| *Baseline* | 1.23 ± 0.49 | 1.22 ± 0.59 |  |  |
| *6^th^ month* | 1.09 ± 0.26 | 1.10 ± 0.26 |  |  |
| **Right DLPFC Activation** | | | **0.829** | **0.054** |
| *Baseline* | 1.12 ± 0.49 | 1.11 ± 0.47 |  |  |
| *6^th^ month* | 1.29 ± 0.94 | 0.92 ± 0.10 |  |  |

* Significant at *p* < 0.05
